# Correlation between variant allele frequency and mean tumor molecules with tumor burden in patients with solid tumors

**DOI:** 10.1002/1878-0261.13557

**Published:** 2023-12-23

**Authors:** Ekaterina Kalashnikova, Vasily N. Aushev, Allyson Koyen Malashevich, Antony Tin, Shifra Krinshpun, Raheleh Salari, Carly Bess Scalise, Rosalyn Ram, Meenakshi Malhotra, Harini Ravi, Himanshu Sethi, Stephanie Sanchez, Robert Tanner Hagelstrom, Maxim Brevnov, Matthew Rabinowitz, Solomon Moshkevich, Bernhard G. Zimmermann, Minetta C. Liu, Alexey Aleshin

**Affiliations:** ^1^ Natera, Inc. Austin TX USA

**Keywords:** biomarkers, circulating tumor DNA, mean tumor molecules, variant allele frequency

## Abstract

Several studies have demonstrated the prognostic value of circulating tumor DNA (ctDNA); however, the correlation of mean tumor molecules (MTM)/ml of plasma and mean variant allele frequency (mVAF; %) with clinical parameters is yet to be understood. In this study, we analyzed ctDNA data in a pan‐cancer cohort of 23 543 patients who had ctDNA testing performed using a personalized, tumor‐informed assay (Signatera™, mPCR‐NGS assay). For ctDNA‐positive patients, the correlation between MTM/ml and mVAF was examined. Two subanalyses were performed: (a) to establish the association of ctDNA with tumor volume and (b) to assess the correlation between ctDNA dynamics and patient outcomes. On a global cohort, a positive correlation between MTM/ml and mVAF was observed. Among 18 426 patients with longitudinal ctDNA measurements, 13.3% had discordant trajectories between MTM/ml and mVAF at subsequent time points. In metastatic patients receiving immunotherapy (*N* = 51), changes in ctDNA levels expressed both in MTM/ml and mVAF showed a statistically significant association with progression‐free survival; however, the correlation with MTM/ml was numerically stronger.

## Graphical abstract

Several studies have demonstrated the prognostic value of ctDNA, with mean tumor molecules (MTM)/ml and mean variant allele frequency (mVAF, %) as two validated metrics for quantifying tumor burden. However, the agreement between these two metrics with clinical parameters is yet to be understood. Here, we assessed the performance of mVAF and MTM/ml for predicting survival after definitive treatment.

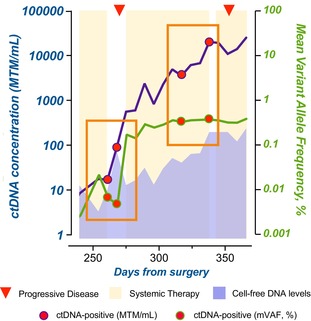

**Abbreviations**
cfDNA: cell‐free DNA
CLIA: Clinical Laboratory Improvement Amendments
ctDNA: circulating tumor DNA
ddPCR: digital droplet PCR
HR: hazard ratio
ICI: immune checkpoint inhibitors
IO: immunotherapy
IRB: institutional review board
mPCR: multiplex PCR
MRD: minimal/molecular residual disease
MTM: mean tumor molecules
mVAF: mean variant allele frequency
NCCN: National Comprehensive Cancer Network
NGS: next‐generation sequencing
NTC: no‐template control
PD: progressive disease
QC: quality control
SD: stable disease
SNV: single nucleotide variants
WES: whole exome sequencing

## 1 Introduction

Measuring circulating tumor DNA (ctDNA) has emerged in recent years as a powerful minimally invasive tool thought to reflect disease burden in cancer patients [1]. Monitoring changes in ctDNA levels over time can serve as a dynamic biomarker for tumor responses to radiation and systemic therapy, as well as detection of minimal/molecular residual disease (MRD) in the postsurgical setting. Because ctDNA can be assessed in a minimally invasive way through a simple blood draw, it is possible to measure it frequently, as compared to standard‐of‐care, radiological imaging, which is typically recommended every 2–3 months in high‐risk patients [2, 3, 4, 5, 6, 7]. These unique capabilities of ctDNA have resulted in a paradigm shift in the assessment of recurrence risk and disease management in cancer patients [8]. In addition, the measurement of ctDNA has informed the design of a growing number of clinical studies [1, 9].

Several methodologies have been developed for detection and quantification of ctDNA, including personalized (tumor‐informed) and fixed panel (tumor‐naive) approaches. Generally, ctDNA measurement relies on quantifying tumor‐specific variants within cell‐free DNA (cfDNA). Given cfDNA originates from a variety of sources, including cancer cells and cells from the tumor microenvironment, immune system, and other organs [10], it is critical that ctDNA methodologies identify ctDNA with high sensitivity and specificity. To date, there are two main metrics used to quantify tumor burden in the blood: mean variant allele frequency (mVAF, %) and mean tumor molecules (MTM) per milliliter of plasma. Mean VAF refers to the percentage of tumor‐specific (i.e., mutated) DNA copies to somatic (i.e., nonmutated) DNA copies [11]. Mean VAF is typically represented as a percentage or proportion and does not provide a concentration of tumor‐specific variants. Thus, when mVAF is quantified, the value is inversely proportional to the amount of total cfDNA in the blood, assuming cfDNA concentration is stable. In contrast to mVAF, MTM/ml is a metric that takes into account both ctDNA and total cfDNA.

Although MTM/ml and mVAF are both validated metrics for ctDNA, the agreement between these two metrics with reference to clinical truth is yet to be understood. Some reports have demonstrated that ctDNA reported as MTM/ml is associated with tumor burden and survival outcomes [1, 12, 13]. Bos et al. [11] recently performed a head‐to‐head comparison of MTM/ml versus mVAF in 338 patients analyzing 1116 tumor‐specific variants and found that mVAF and MTM/ml were more concordant when blood samples were analyzed by digital droplet PCR (ddPCR) and more discordant when analyzed using next‐generation sequencing (NGS) due to insufficient molecular coverage on NGS and high cfDNA concentration. Here, we examine the concordance between MTM/ml versus mVAF in a larger pan‐cancer cohort, using a personalized and tumor‐informed multiplex (m)PCR NGS‐based ctDNA assay, Signatera™, which employs an amplicon‐based sequencing, with an average depth of read per amplicon of > 105 000×. The study specifically assesses the performance of both mVAF and MTM/ml for predicting patient survival after definitive treatment.

## 2 Materials and methods

### 2.1 Patient cohort

In this retrospective analysis, bio‐banked plasma samples were processed at Natera, Inc. between 5/30/2019 and 10/4/2022. The cohort included: (a) a real‐world commercial dataset of patients who received a primary diagnosis of colorectal cancer, breast cancer, pancreatic cancer, esophageal cancer, gastrointestinal cancer, melanoma, lung cancer, or other cancers. (b) A subanalysis assessed the correlation between ctDNA levels (measured in MTM/ml and mVAF) with imaging in a cohort of patients with high‐risk early‐stage breast cancer disease treated in the neoadjuvant setting [14]. (c) Another subcohort analysis was performed to assess the association between ctDNA dynamics (MTM/ml and mVAF separately) and survival outcomes in patients with advanced solid tumors (i.e., melanoma, upper gastrointestinal, gynecologic, and genitourinary cancers) receiving immunotherapy. Blood samples from commercial cases were collected longitudinally at the discretion of the treating physician. Patient treatment was also administered at the discretion of the physician in accordance with National Comprehensive Cancer Network (NCCN) guidelines. Clinicopathologic information for the real‐world dataset used in the analyses was collected retrospectively from Natera's commercial database under an IRB‐approved protocol (# 20‐049‐ALL) or as a secondary analysis using de‐identified data from the ISPY study. This study is a secondary analysis of the existing data and therefore is exempt from the requirement for written informed consent. This study was conducted in accordance with the Declaration of Helsinki.

### 2.2 Personalized tumor‐informed mPCR‐NGS‐based ctDNA testing

ctDNA was quantified using a personalized and tumor‐informed assay (Signatera™, mPCR‐NGS assay), which is previously described [15, 16]. Briefly, formalin‐fixed and paraffin‐embedded tumor tissue along with matched normal blood samples were subjected to whole exome sequencing (WES). Based on sequencing results, up to 16 patient‐specific, clonal somatic single nucleotide variants (SNVs) were selected, for which primers were designed and synthesized to perform the Signatera assay. Plasma samples isolated from whole blood were then analyzed for ctDNA (median collection = 10 mL of plasma). Samples with at least two variants detected above a predefined algorithmic confidence threshold were defined as ctDNA‐positive. Plasma was collected longitudinally to monitor ctDNA levels in response to treatment.

### 2.3 Calculation of MTM/ml from mean VAF (%) and cfDNA

For plasma samples with a positive call, mean VAF of all the targets is computed and is used in the formula below along with measured plasma volume and measured total cfDNA mass to determine the mean tumor molecules per ml of plasma (MTM/ml).

$$\mathrm{MTM}/\mathrm{ml}=\frac{\text{mean}\;\mathrm{VAF}\;x\;\text{cfDNA mass}\;\left(\mathrm{ng}\right)\times 1000\;\mathrm{pg}/\mathrm{ng}}{3.3\;\mathrm{pg}\times \text{plasma volume}\;\left(\mathrm{mL}\right)}$$

Note: 3.3 pg is the mass of a single haploid copy of the human genome; cfDNA mass is cfDNA quant‐iT concentration × 45.

Below we explain the three inputs into the Signatera assay workflow, which are used for calculating the MTM/ml value. The first two components, namely cfDNA concentration and the plasma volume that are used to calculate MTM, are derived from a process that uses calibrators. The third component, VAF as mentioned is a ratio (measured using NGS data as ratio of mutant reads to reference reads) and is specific to every patient. Additionally, for reporting MTM/ml values, the Signatera process uses four controls: no‐template control (NTC), negative, and two positive controls that have low and high MTM/ml readout to ensure the process robustness for reporting MTM/ml.

- *cfDNA concentration*: The cfDNA concentration is measured in ng·μL^−1^. For every batch of Signatera CLIA samples, cfDNA quantity is measured by an off‐the‐shelf quantification kit named Quant‐iT™ dsDNA Assay Kit, High Sensitivity (Life Technology Corporation, Eugene, OR, USA). A total of eight standards of concentrations ranging from 0 to 10 ng·μL^−1^ are available in the kit. The kit uses a calibration curve generated from the eight standards, and the concentrations of Signatera samples are then derived based on the calibration curve. The cfDNA concentration measurements are derived based on the calibration standards run in every batch. These standards are qualified for Signatera CLIA use per lot using an independent QC process. For Quant‐iT measurements that fall above the calibration standards (> 10 ng·μL^−1^), cfDNA concentration is extrapolated based on the standard curve. The majority of Signatera samples fall within the 0–10 ng·μL^−1^ range, accounting for approximately the 98^th^ percentile of Signatera CLIA samples. The cfDNA concentration as measured by Quant‐iT is multiplied by 45 μL to achieve the cfDNA mass (ng). The cfDNA mass is then divided by the plasma volume (mL) to get cfDNA concentration in ng·mL^−1^.
- *Plasma Volume*: Plasma volume is measured by using an automated liquid level reader on the Tecan instrument. Volume is calculated by measuring liquid level, separation level, and tube height. Repeat plasma volume measurements are highly precise with a coefficient of variance in the range of 1% and have been internally validated.
- *Percentage VAF*: Several post‐sequencing quality control (QC) metrics are applied to each sample prior to running the variant calling algorithm. The proprietary Signatera algorithm makes a positive or negative call for each target. VAF for each target is calculated as the ratio of mutant reads to reference reads. Mean VAF is calculated as the average measured VAF of all QC‐passing Signatera targets (where negatively called targets are set to 0 VAF). Mean VAF is calculated only for positively called samples. VAF is a ratio derived for each sample, using targets unique to that sample. It cannot be normalized to or derived from an external batch‐level calibration curve. In order to normalize results obtained over time and to describe in terms of the ctDNA analyte as a whole, Signatera reports the results in MTM/ml as a crucial metric over VAF.

### 2.4 Statistical analysis

The relationship between the MTM/ml and mVAF was modeled using LOESS regression. The association of MTM/ml, mVAF, and tumor volume was assessed by Pearson correlation. Variables were assessed for normality and a log10 transformation was applied prior to correlation analysis. Correlations were compared using William's test [17]. For the survival analysis, the primary outcome was overall survival. Survival analyses were performed using the Kaplan–Meier Estimator and the Cox method. These analyses were carried out in stata version 16.1 (StataCorp, College Station, TX, USA). All *P*‐values were based on two‐sided testing, and differences were considered significant at *P* ≤ 0.05.

## 3 Results

### 3.1 Correlation between MTM/ml and mean VAF (%)

**Fig. 1:**
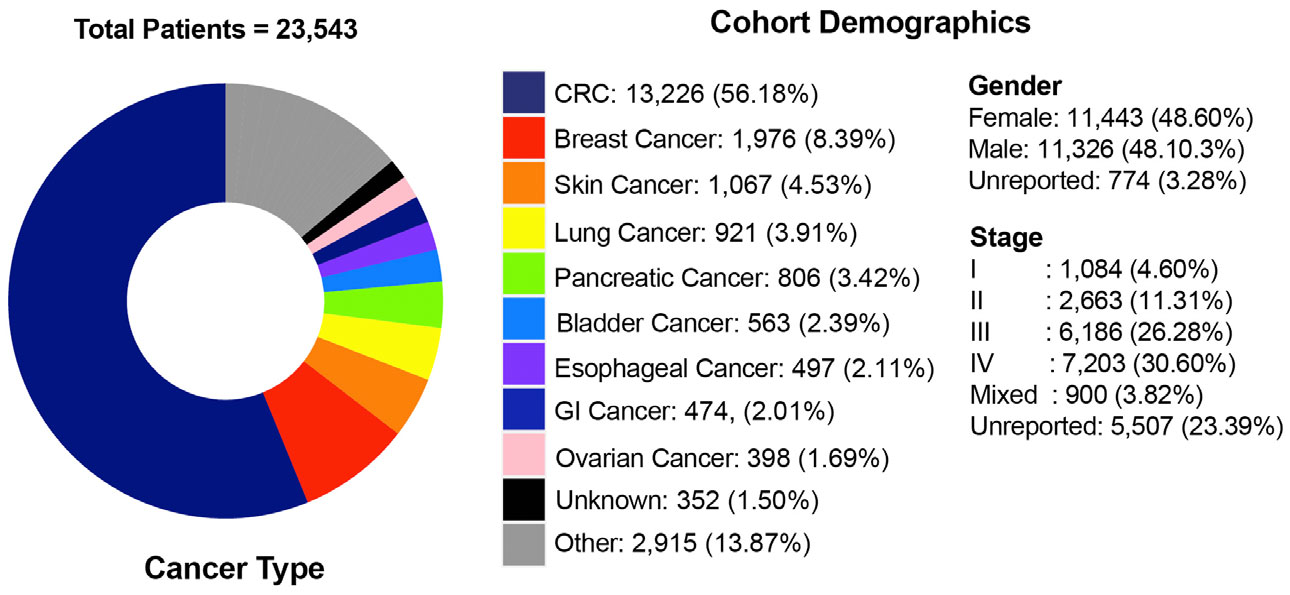
Cohort characteristics. Breakdown of cancer types in 23 543 patients with solid tumors. CRC, colorectal cancer; GI, gastrointestinal.

We first examined the correlation between MTM/ml and mVAF in a commercial CLIA cohort. A total of *N* = 23 543 patients (*n* = 91 562 blood samples) with at least one ctDNA‐positive result (*n* = 55 183) were included in this analysis. The primary diagnosis of the patients included: colorectal cancer (*N* = 13 226), breast cancer (*N* = 1976), skin (*N* = 1067), lung cancer (*N* = 921), pancreatic cancer (*N* = 806), bladder (*N* = 563), esophageal cancer (*N* = 497), gastrointestinal cancer (*N* = 474), ovarian cancer (*N* = 398), other cancers (*N* = 2915), and cancers of unknown or not reported histology (*N* = 352; Fig. 1). Of the patients included, 18 426 patients had longitudinal (more than one time point) plasma samples (*n* = 86 445) available with at least one ctDNA‐positive time point.

**Fig. 2:**
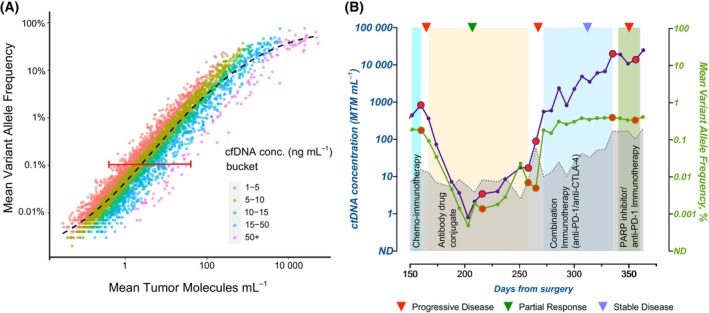
Correlation between MTM/ml and mean VAF (%). (A) Correlation of MTM/ml versus mean VAF (%) across ctDNA‐positive samples. Samples bucketed by total cfDNA concentration (ng·mL^−1^) are represented. Red line indicates linear correlation between MTM/ml and mean VAF (%) (log_meanVAF_ = 0.87 log_MTM/ml_ − 7.58; R^2^ = 0.932). Black line indicates LOESS regression. As represented, when MTM/ml and VAF were compared based on cfDNA, at >50 ng·mL^−1^ (pink dots), a wide horizontal distribution of MTM/ml is observed for any given mVAF value. (B) Utility of MTM/ml versus mean VAF (%) in informing treatment decisions in a patient with metastatic triple‐negative breast cancer. During treatment response monitoring with ctDNA, serial (longitudinal) time points were processed at regular intervals. ctDNA measured in MTM/ml (purple line) or mean VAF (%) (green line) is represented. Changes in ctDNA dynamics from the previous time point were tracked, and discrepancies in ctDNA dynamics as measured by MTM/ml versus mean VAF (%) were noted (red dots). Imaging results as defined by RECIST criteria are indicated by inverted triangles. Gray line indicates cfDNA measurement. Adapted with permission from Azzi et al. 2022, Case Reports in Oncology, 2022;15:473–479, Published by S. Karger AG, Basel. cfDNA, cell‐free DNA; ctDNA, circulating tumor DNA; MTM, mean tumor molecules; VAF, variant allele frequency.

Among the pan‐cancer cohort of 23 543 patients with 55 183 ctDNA‐positive blood samples, the median cfDNA concentration was 6.9 ng·mL^−1^ (range: 0.46–937.18 ng·mL^−1^). Regression analysis revealed strong correlation between mVAF and MTM/ml (linear regression: log_meanvAF_ = 0.87 log_MTM/ml_ − 7.59, *R* ^2^ = 0.932; LOESS regression: *R* ^2^ = 0.940, red line; Fig. 2A). At <5 ng·mL^−1^ (red dots) and > 50 ng·mL^−1^ (pink dots) of cfDNA concentration accounting for 1896 plasma samples, a wide (horizontal) distribution for MTM/ml values were observed for a given mVAF value. Additionally, MTM/ml displayed a continuous dynamic range at around 10 000 MTM/ml, while we observed mVAF values plateauing at 100% (Fig. 2B). Taken together, although our correlation analysis reveals that MTM/ml and mVAF are generally well correlated, ctDNA quantified in MTM/ml may be more representative of tumor burden for a broader range of values. Furthermore, when considering ctDNA‐positive patients who had serial samples collected (*N* = 18 426), 13.3% of patients (2450/18 426) were assessed to have discordant results between MTM/ml and mVAF dynamics, that is, between any two subsequent time points, an increase in MTM/ml value corresponded with a decrease in mVAF value or vice versa.

### 3.2 Clinical utility of MTM/ml versus mean VAF (%) in guiding treatment in a case study of a patient with mTNBC

We next asked whether differences in MTM/ml and mVAF could impact physician decision‐making over the course of treatment in individual patients. We assessed ctDNA dynamics by MTM/ml and mVAF in a triple‐negative breast cancer (TNBC) patient, who underwent longitudinal ctDNA testing (Fig. 2B) [18]. In this particular case, the MTM/ml and mVAF followed a similar trajectory; though not exclusive, the two metrics were observed to be more frequently discordant (denoted as red dots) at the beginning or conclusion of therapy. Such discordances are typically observed in instances when background cfDNA is elevated, likely due to the change in therapy regimen or other physiological factors. In all marked (red dots) instances, mVAF failed to reflect an increase in ctDNA, while MTM/ml values corresponded with disease progression on imaging. For example, toward the end of 4^th^ line therapy (PARP inhibitor/anti‐PD‐1 immunotherapy), the patient's molecular profile by mean VAF (%) suggested stable disease (SD), while MTM/ml suggested disease progression. Although imaging results at day 325 were in line with SD, by day 350, imaging identified progressive disease (PD). Thus, MTM/ml was able to detect disease progression ahead of radiological findings, while mVAF was not. Accurate and timely measurement of tumor dynamics at critical time points during treatment may have a significant impact on clinical decision outcomes.

### 3.3 Performance of MTM/ml versus mean VAF (%) in assessing clinical outcomes

Next, we examined the relationship between MTM/ml and mVAF with clinical characteristics. In a cohort of early‐stage breast cancer patients (*N* = 103) who had matched pretreatment and on‐treatment time points available, ctDNA levels were correlated with tumor volume measured by imaging. At the pretreatment and on‐treatment time points both MTM/ml and mVAF measurements showed a similar degree of correlation with tumor volume on the log10 scale [(Pretreatment MTM/ml: *r* = 0.49, *P* < 0.0001; mVAF: *r* = 0.51, *P* < 0.0001; Fig. S1a,b; on treatment MTM/ml: *r* = 0.55, *P* < 0.0001; mVAF: *r* = 0.51, *P* < 0.0001), Fig. S1c,d]. Notably, the comparison of the strength of the association between the two metrics and tumor volume did not result in statistically significant findings (thus null hypothesis could not be refuted). Per our estimates, it is unlikely that the observed difference between the two metrics in the presurgical setting would be found statistically significant in a cohort of fewer than 700 cases, while statistical significance may be reached if the same effect size is observed in the on‐treatment setting in a marginally larger cohort (*N* = 193). As the observed trend could impact the interpretation of ctDNA results in the on‐treatment setting, this result requires further validation in a larger sample set (Table S1). We next examined the effect of systemic therapy on cfDNA levels. For patients with cfDNA results available at pretreatment (*N* = 103) and on‐treatment (*N* = 103) time points, we observed cfDNA levels to be elevated during treatment compared with pretreatment (*P* < 0.0001; Fig. S1e).

**Fig. 3:**
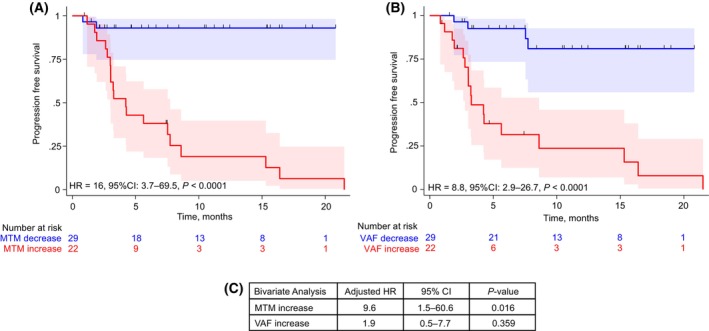
Prognostic value of stratifying patients by MTM/ml or mean VAF (%) to predict treatment response in metastatic patients treated with IO. Patients diagnosed with metastatic melanoma (*n* = 22), upper gastrointestinal (*n* = 14), gynecologic (*n* = 11), and genitourinary (*n* = 4) cancers with longitudinal ctDNA testing (*N* = 51) were stratified by increase or no increase in ctDNA, measured in: (A) MTM/ml, and (B) mean VAF (%). (C) Bivariate analysis to assess the predictive value of MTM and mean VAF dynamics. Survival analyses were performed using the Kaplan–Meier Estimator and the Cox method. ctDNA, circulating tumor DNA; IO, immunotherapy; MTM, mean tumor molecules; VAF, variant allele frequency.

We next sought to evaluate MTM/ml vs. mVAF dynamics to predict patients' response to treatment and survival. Patients with metastatic pan‐cancer disease (*N* = 51) undergoing treatment with an immune checkpoint inhibitor (ICI) who had a ctDNA increase had poorer survival outcomes as compared to those with no increase in ctDNA. Interestingly, although changes in ctDNA determined by both metrics showed a statistically significant association with patient survival, the correlation with MTM/ml was numerically stronger (ctDNA by MTM/ml: Hazard Ratio (HR): 16, 95% CI: 3.72–69.5, *P* < 0.0001; ctDNA by mVAF HR: 8.8, 95% CI: 2.9–26.7, *P* < 0.0001; Fig. 3A,B). Furthermore, the bivariate model dynamics in MTM/ml were observed to be an independent and significant predictor of outcome when compared with mVAF (Fig. 3C). Eight patients (~ 16%) had discordant ctDNA dynamics when expressed as MTM/ml vs mVAF. Interestingly in 6 (~ 12%) of these patients, mVAF results did not correlate with response by RECIST imaging, while MTM/ml values did not match outcome in only 2 (~ 4%) of the patients from this cohort. Taken together, ctDNA measured in MTM/ml was more predictive of tumor volume and prognostic of overall survival than ctDNA measured in mVAF.

## 4 Discussion

Given the increasing clinical adoption of ctDNA to measure MRD and response to therapy, there is a clear need for ctDNA quantitation that reliably reflects disease status. In our study, we hypothesized that MTM/ml is more reflective of the true physiological stage of the tumor and is more predictive of patient survival outcomes than mVAF, particularly in situations where cfDNA is elevated. This is because VAF is a fraction that is subject to changes in the background cfDNA (the denominator), which can fluctuate based on other biological factors not specific to a patient's disease. Whereas, MTM/ml normalizes for the background cfDNA, to provide the true concentration of tumor DNA in the blood. Especially when performing serial ctDNA testing to evaluate a patient's response to therapy, it would not be appropriate to monitor ctDNA using VAF, since it is fundamentally limited at 100%. Meanwhile, MTM/ml is a highly dynamic marker that generally moves logarithmically. Thus, differences between MTM/ml and mVAF can have an impact on patient's treatment decisions related to the initiation, continuation, or cessation of therapy, ahead of radiological findings.

The observed discrepancies between MTM/ml and mVAF are best explained by elevation in cfDNA. It has been reported that mVAF can be confounded at high concentrations of cfDNA [11]. In contrast, the MTM/ml calculation is a metric that accounts for cfDNA. The increase in cfDNA levels can be associated with a number of factors, both specific to and independent of cancer, such as the presence of metastatic disease, tissue damage due to cytotoxic therapies, after surgery and during inflammation [19, 20] as well as age, gender, diet, smoking, level of activity, glucose levels, oxidative stress, tissue trauma/surgery, pregnancy, renal function, and autoimmune disease [19, 21]. Many of these are difficult to account for. Thus, the use of mVAF in a high cfDNA background may lead to test results with lower accuracy. Conversely, ctDNA measured in MTM/ml accounts for cfDNA levels in its calculation, making it a more reliable measure of tumor burden. Among the 13% (2450/18 426) of discordant cases observed in our study, a representative case as illustrated in Fig. 2B demonstrates the discrepancy between MTM/ml and mVAF dynamics at critical time points, especially at the beginning and the conclusion of the therapy. Importantly, ctDNA dynamics measured in MTM/ml were observed to more closely correspond with imaging results showing PD and can therefore provide better guidance for physician decision‐making.

Of note, it should be considered that, as a derived quantity, MTM/ml consists of three separate measurement procedures, that is, cfDNA concentration, plasma volume, and percentage mVAF, each with their own inherent measurement variability. Factors that can introduce technical variability include but are not limited to efficiency of DNA extraction from plasma, machine‐to‐machine variability in measurement of DNA in plasma, efficiency of ctDNA primers, sequencing coverage, and amplification of mutant versus reference DNA occurring at different levels of efficiency [22, 23, 24].

In our correlative analysis (Fig. 2), we observed a positive correlation between MTM/ml and mVAF that followed the line of best fit at low and moderate levels of ctDNA. For example, at a given value of mVAF (0.1%), we observed a wide horizontal range of MTM/ ml values (range: < 1 to > 50 MTM/ml), depending on the variable levels of total cfDNA (< 5 to > 50 ng·mL^−1^) in the sample (Fig. 2A). In the case presented in Fig. 2B, at ctDNA levels > 10 000 MTM/ml, while MTM/ml continues to display a dynamic range of values across samples, mVAF measurements are maximized at 100% saturation. This observation suggests that at higher cfDNA concentrations, which are frequently observed during the active phase of treatment, mVAF may not be as reliable in reflecting the true disease status as MTM/ml, for guiding clinical decision‐making. Only one other study to date has examined the correlation between MTM/ml and mVAF, where a mixed cohort of 338 patients with ctDNA measured by either NGS or ddPCR. They found that MTM/ml and mVAF were well correlated when ctDNA was assessed by ddPCR and that insufficient molecular coverage by NGS or high background cfDNA resulted in greater discordance between MTM/ml and mVAF [11]. In contrast to our study, correlations with clinical outcomes were not performed.

These preliminary findings suggest that MTM/ml may be more accurate in assessing clinical parameters such as tumor volume and response to treatment by imaging, compared with mVAF. However, these findings would need to be validated in a larger cohort. Previous work correlating ctDNA in MTM/ml and tumor volume has been reported [14]. Specifically, in our subcohort analysis of early‐stage breast cancer patients, we observed that though mVAF produced an accurate estimation of tumor fraction in the pretreatment setting, it may be less reliable during the active phase of treatment, likely due to the mechanism of action of the drugs that lead to cell‐death contributing to elevated cfDNA levels. This results in a decrease in the numeric value of mVAF (tumor fraction) that is not always consistent with disease burden. While calculating MTM/ml, on the contrary, an increase in the background cfDNA (nonspecific to tumor) is taken into account and the resulting estimate is more representative of the clinical disease status. Overall, our data suggest that both mVAF and MTM/ml are validated metrics for measuring ctDNA concentration; MTM/ml would be more representative of measuring molecular disease burden, specifically in scenarios that lead to an increase in cfDNA levels. Our data are currently limited by sample size and the weak nature of the association of either of the metrics with tumor volume. Thus, a larger dataset would be needed to validate these findings (Table S1).

Several studies have laid the foundation for the prognostic evaluation of ctDNA measured as an absolute concentration (MTM/ml) or as a proportion mVAF. In terms of ctDNA as an absolute measurement, Seremet et al. found that in a cohort of 63 metastatic melanoma patients treated with anti‐PD‐1 therapy, the presence of ctDNA at baseline after treatment, measured in absolute value (ctDNA copies per ml of plasma) was associated with poorer median progression‐free survival [26 weeks (95% CI: 0–71.1) vs. 9 weeks (95% CI: 6.9–22; *P* = 0.008)] and median overall survival (21 weeks versus not reached; 95% CI: 0–43; *P* = 0.003) [25]. Lecomte et al. similarly demonstrated that absolute ctDNA was predictive of overall survival (48%, as compared to 100% for patients without ctDNA (*P* < 0.03)) in patients with colorectal cancer [26]. Other studies have reported on the prognostic value of ctDNA measured using mVAF [27, 28]. Our study is the first to directly compare the ability of MTM/ml versus mVAF to predict clinical outcomes. In our survival analysis of a subcohort of patients with advanced cancer treated with immunotherapy, results suggest that MTM/ml is numerically stronger in predicting progression‐free survival than mVAF (Fig. 3). Approximately 12% of patients were misclassified by mVAF (%) compared to 4% misclassified by MTM/ml, which can misinform treatment decisions and lead to heightened patient anxiety.

## 5 Conclusions

Given the wide acceptance and emerging utility of ctDNA in various cancer indications for detecting molecular residual disease and monitoring response to therapy, there exists a definite need for a ctDNA metric that accurately reflects disease burden. Though MTM/ml and mVAF are both validated metrics for ctDNA measurement, our study suggests that MTM/ml could be more representative of molecular disease burden. Specifically, in patients with metastatic disease receiving immunotherapy, ctDNA dynamics expressed in MTM/ml were more predictive of patient outcomes. Thus, we conclude that ctDNA quantification and dynamics measured in MTM/ml may improve correlation with clinical parameters when used as the unit of measurement for ctDNA.

## Conflict of interest

AKM is a former employee of Natera, Inc. and holds stock in the company. All other authors are full‐time employees of Natera, Inc. and have stock/options to own stock in the company. MCL: grants/contracts: funding to Institution (Mayo) from Eisai, Exact Sciences, Genentech, Genomic Health, GRAIL, Menarini Silicon Biosystems, Merck, Novartis, Seattle Genetics, and Tesaro; travel support reimbursement from AstraZeneca, Genomic Health, and Ionis; ad hoc advisory board meetings. All funds to Mayo Clinic. No personal compensation from AstraZeneca, Celgene, Roche/Genentech, Genomic Health, GRAIL, Ionis, Merck, Pfizer, Seattle Genetics, Syndax. MR: Stock for Myome, GenEdit, Algen Bio, Conceptions, Themba, and Marble Therapeutics; Leadership/fiduciary role in ACMG Foundation; Royalties/Licenses for Natera, Inc.

## Author contributions

EK, VNA, and SS performed statistical analysis for the study. AT, SK, and CBS contributed to data abstraction and interpretation. RR, HR, and HS contributed to the methodological details. AKM and MM wrote the first draft of the manuscript and interpreted the results. RS, HR, RTH, MB, HS, MR, SM, BGZ, MCL, and AA contributed to the conceptualization and design of the study. AA contributed to overall study supervision and administration. All other authors provided a thorough review of the analysis and the manuscript. All authors approved the final draft of the manuscript.

## Supporting information


**Fig. S1.** Correlation between MTM/ml or mean VAF (%) and tumor volume.
**Table S1.** Correlation between MTM/ml or mean VAF (%) and tumor volume.

## Data Availability

All data generated during this study are included in this article. Further enquiries can be directed to the corresponding author.
