# Supplementary material for: Correlation between variant allele frequency and mean tumor molecules with tumor burden in patients with solid tumors
Source: Mol Oncol. 2023 Dec 23;18(11):2649–57. doi: 10.1002/1878-0261.13557 (PMC11547219; doi:10.1002/1878-0261.13557)
Supplement: Supplementary file 1 — Fig. S1. Correlation between MTM/ml or mean VAF (%) and tumor volume. Table S1. Correlation between MTM/ml or mean VAF (%) and tumor volume. [file MOL2-18-2649-s001.zip › Supporting Information-MTM vs VAF- Dec21-2023.docx]

**Supporting Information**


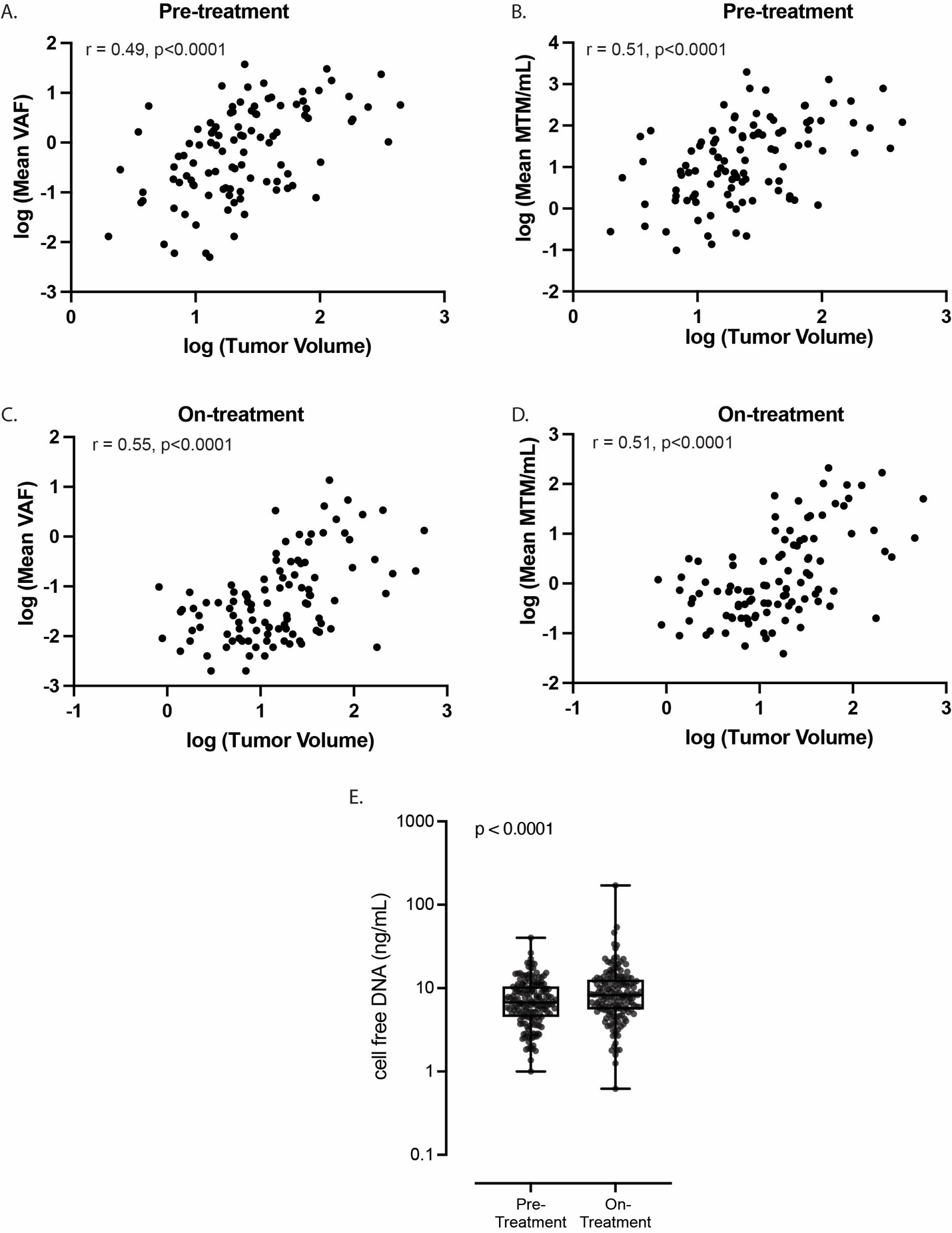


**Supplementary Figure 1. Correlation between MTM/ml or mean VAF (%) and tumor volume.**

**A-B.** Tumor volume in early-stage breast cancer patients with longitudinal ctDNA testing (only positive ctDNA samples were included; N=103) was plotted against mean ctDNA values as measured by **(A)** MTM/ml or **(B)** mean VAF (%)**. C.** Early-stage breast cancer patients with cfDNA were stratified by treatment status (regardless of ctDNA status) (pre-treatment, N=103 versus on-treatment, N=103). Total cfDNA levels (ng/ml) were significantly different between the two groups (pretreatment: median 6.7 (6.2-7.6) vs on-treatment: 8.3 (7.4-8.9), p<0.0001). Each sample corresponds to an individual patient. Association of MTM/ml, mVAF, and tumor volume was assessed by Pearson correlation. Variables were assessed for normality and a log10 transformation was applied prior to correlation analysis. Correlations were compared using William’s test. Abbreviations: MTM, mean tumor molecules; VAF, variant allele frequency; ctDNA, circulating tumor DNA; cfDNA, cell-free DNA

**Supplementary Table 1. Correlation between MTM/ml or mean VAF (%) and tumor volume.**

| **Timepoint** | **Comparison** | **Pearson** | **Difference 95% CI** | **Spearman** | **Pearson of Log10 Values** | **Difference 95% CI** | **N Needed to Claim Significance At This Effect Size** |
| --- | --- | --- | --- | --- | --- | --- | --- |
| Pre-Treatment | MTM vs MRI | .20 (0.0395) | (-0.18, -0.04) | .50 (<0.0001) | .49 (<0.0001) | (-0.08, 0.04) | 728 |
|  | VAF vs MRI | .31 (0.0016) |  | .52 (<0.0001) | .51 (<0.0001) |  |  |
|  | MTM vs VAF | .94 (<0.0001) |  | .95 (<0.0001) | .95 (<0.0001) |  |  |
| On-Treatment | MTM vs MRI | .25 (0.0109) | (0.02, 0.18) | .51 (<0.0001) | .55 (<0.0001) | (-0.02, 0.10) | 193 |
|  | VAF vs MRI | .15 (0.1275) |  | .47 (<0.0001) | .51 (<0.0001) |  |  |
|  | MTM vs VAF | .91 (<0.0001) |  | .94 (<0.0001) | .95 (<0.0001) |  |  |
